# Adhesive Interactions Between Lactic Acid Bacteria and β-Lactoglobulin: Specificity and Impact on Bacterial Location in Whey Protein Isolate

**DOI:** 10.3389/fmicb.2019.01512

**Published:** 2019-07-03

**Authors:** Faustine Gomand, Frédéric Borges, Justine Guerin, Sofiane El-Kirat-Chatel, Gregory Francius, Dominique Dumas, Jennifer Burgain, Claire Gaiani

**Affiliations:** ^1^Laboratoire d’Ingénierie des Biomolécules, Université de Lorraine, Vandœuvre-lès-Nancy, France; ^2^CNRS, Laboratoire de Chimie Physique et Microbiologie pour les Matériaux et l’Environnement (LCPME), UMR 7564, Université de Lorraine, Villers-lès-Nancy, France; ^3^Plateforme d’Imagerie et de Biophysique Cellulaire de Nancy (PTIBC IBISA-NANCY), UMS 2008, IMOPA UMR 7365 - Université de Lorraine, Vandœuvre-lès-Nancy, France

**Keywords:** adhesion, lactic acid bacteria, dairy, β-lactoglobulin, high-throughput screening, bacterial distribution, atomic force microscopy (AFM), confocal laser scanning microscopy (CLSM)

## Abstract

In the last decade, there has been an increasing interest in the potential health effects associated with the consumption of lactic acid bacteria (LAB) in foods. Some of these bacteria such as *Lactobacillus rhamnosus* GG (LGG) are known to adhere to milk components, which may impact their distribution and protection within dairy matrices and therefore is likely to modulate the efficiency of their delivery. However, the adhesive behavior of most LAB, as well as its effect on food structuration and on the final bacterial distribution within the food matrix remain very poorly studied. Using a recently developed high-throughput approach, we have screened a collection of 73 LAB strains for their adhesive behavior toward the major whey protein β-lactoglobulin. Adhesion was then studied by genomics in relation to common bacterial surface characteristics such as pili and adhesion-related domain containing proteins. Representative adhesive and non-adhesive strains have been studied in further depth through biophysical measurement using atomic force microscopy (AFM) and a relation with bacterial distribution in whey protein isolate (WPI) solution has been established. AFM measurements have revealed that bacterial adhesion to β-lactoglobulin is highly specific and cannot be predicted accurately using only genomic information. Non-adhesive strains were found to remain homogeneously distributed in solution whereas adhesive strains gathered in flocs. These findings show that several LAB strains are able to adhere to β-lactoglobulin, whereas this had only been previously observed on LGG. We also show that these adhesive interactions present similar characteristics and are likely to impact bacterial location and distribution in dairy matrices containing β-lactoglobulin. This may help with designing more efficient dairy food matrices for optimized LAB delivery.

## Introduction

Adhesion is a major property of microorganisms which effectively impacts microorganism activities as well as human health, and has been identified as a key factor involved in microorganism ecology. Adhesion enables bacteria to stick to both biotic and abiotic surfaces. Adhesion to abiotic surfaces leads to biofilm formation, which has been widely studied in relation to the food industry (Notermans et al., 1991; Pontefract, 1991; Barnes et al., 2001; Garrett et al., 2008). Adhesion to biotic surfaces enables bacteria to establish direct contact with mucous membranes, and especially the intestinal epithelium, to colonize a host (Conway et al., 1987; Servin and Coconnier, 2003; Pizarro-Cerdá and Cossart, 2006). Adhesion of pathogens is therefore considered to be a virulence factor as it facilitates host invasion (Pizarro-Cerdá and Cossart, 2006; Proft and Baker, 2009). Amongst non-pathogenic bacteria, adhesion is considered essential in order for probiotic bacteria to remain functional and therefore provide health benefits to the host (Ouwehand et al., 2001; Servin and Coconnier, 2003; Quinto et al., 2014). In the case of gram-positive bacteria, bacteria-environment interactions such as bacterial adhesion are mediated by sortase-dependent proteins (Comfort and Clubb, 2004; Maresso and Schneewind, 2008), which are covalently anchored to the cell wall and possess an LPxTG like motif at their C-terminal end (Schneewind and Missiakas, 2014).

Bacteria have also been shown to be able to adhere to food components, especially to meat (Firstenberg-Eden, 1981; Piette and Idziak, 1989) and more recently to dairy components (Burgain et al., 2014a; Guerin et al., 2016; Gomand et al., 2018). Bacterial adhesive interactions to food components can compete with bacterial adhesion to the host (Sun and Wu, 2017). Therefore food components such as milk fat globule membrane (Douëllou et al., 2017; Guerin et al., 2018b), milk proteins (Halpin et al., 2008), and milk oligosaccharides (Lane et al., 2012) can play an anti-adhesive role by decreasing bacterial adhesion to the intestine (Guerin et al., 2018b). Some food additives including stabilizers (such as sucrose fatty acid esters) and colors (gardenia yellow, monascus pigment, etc.) have also been found to feature similar effects (Islam et al., 2014).

In food matrices, adhesive interactions are likely to play an important part in bacterial spatial distribution and viability during the structuration of the food matrix (Gomand et al., 2019). Adhesive interactions occurring between the model strain *Lactobacillus rhamnosus* GG (LGG) and β-lactoglobulin is mediated by the pili produced by LGG cells on their surface (Guerin et al., 2016). These interactions result in an increased encapsulation efficiency when using dairy components as well as a higher resistance to gastric digestion for this strain (Burgain et al., 2013a, 2014b; Guerin et al., 2017). Adhesive interactions between genetically engineered *Lactococcus lactis* producing pili and dairy components result in texture alteration in fermented milk (Tarazanova et al., 2018a) and can modulate this strain distribution in cheese curd (Tarazanova et al., 2018b). Similarly, during curdling and cheese ripening, bacterial cells mostly co-localize with fat globules or at the casein-fat interface, which suggest adhesive interactions between fat and lactic acid bacteria (LAB) strains (Laloy et al., 1996; Lopez et al., 2006). This is likely to play a role in lipolysis thus affecting the development of characteristic flavors and textures during ripening (Laloy et al., 1996; Lopez et al., 2006).

However, the impact and technological interest of adhesive interactions is yet poorly documented and largely remains to be investigated (Hickey et al., 2015). Adhesive interactions between bacterial surface components and dairy components have only been studied for very few wild type strains, namely LGG (Guerin et al., 2016), *Lactobacillus amylovorus* (Chumphon et al., 2016), and *Lactobacillus paracasei* (De Bellis et al., 2010). This article goes one step forward in that direction by applying the high-throughput screening method recently developed by Gomand et al. (2018) to a collection of 73 LAB strains (for which genome sequence is available) in order to characterize their potential adhesive behavior toward the major dairy protein β-lactoglobulin, to which the adhesive behavior of the model strain LGG is already well-known (Burgain et al., 2013b, 2014b, 2015; Guerin et al., 2016, 2018a). Two strains featuring extreme adhesive and non-adhesive behaviors have then been studied in further depth through atomic force microscopy (AFM). The AFM results were then studied in relation to confocal laser scanning microscopy (CLSM) experiments, allowing to observe the spatial distribution of these strains in whey protein isolate (WPI) solution.

## Materials and Methods

### High-Throughput Screening

Adhesive interactions between bacteria and β-lactoglobulin were screened using the method recently developed by Gomand et al. (2018) using an automated liquid handling system for 96-well microplates.

Briefly, this method consists in immobilizing the biomolecules of interest on the surface of 96 well adherent microplates. Microplates are then washed with a blocking agent in order to remove all unbound molecules and to block the remaining empty sites. The bacterial suspension is then added into the wells and incubated for 1 h at 37°C in order to allow bacterial adhesion to the immobilized biomolecules. Non-adherent bacteria are removed by successive washes using the same blocking agent. The amount of immobilized bacteria is measured through bacterial growth monitoring (turbidity measurements at 595 nm) after the addition of MRS culture growing medium (De Man et al., 1960) in the wells. The higher the initial quantity of bound bacteria, the earlier the growth starts. Adjustments made to this protocol are listed below.

#### Bacterial Strains and Cultures

A list of the 73 screened LAB strains is given in Supplementary Data S1. This collection of strains has previously been studied for their genomics and surface properties (Sun et al., 2015). The model strain LGG ATCC53103 (LGG wild type, “WT”) and the mutant strain LGG *spaCBA* CMPG 5357 impaired in pili synthesis, which adhesive properties of both are well-known (Lebeer et al., 2012; Tripathi et al., 2012, 2013; Guerin et al., 2016) were respectively used as positive (adherent) and negative (non-adherent) control strains.

For each series of experiments, a 96-well microplate previously stored at −80°C was thawed and replicated on working microplates using 50 μL of bacterial suspension to inoculate 150 μL of MRS by well. The working microplates were incubated at 30°C 2 days before the adhesion assay. During the adhesion assay, microplates were only centrifuged once at 1,642 × *g* for 20 min, emptied and the resulting cell pellets were resuspended in 200 μL of PBS adjusted at pH 6.8. Triplicates on independent cultures were performed as well as duplicates by strain on each plate (six repetitions for control strains).

#### Preparation of the β-Lactoglobulin Solution and Microplate Coating

Beta-lactoglobulin (Sigma-Aldrich Co. LLC, St Louis, MO, United States) was prepared in solution (1% w/w) as described by Gomand et al. (2018).

#### Bacterial Growth Monitoring

Adhesion and growth monitoring were done according to Gomand et al. (2018). The incubation temperature was changed to 30°C in order to match the diversity of the growing conditions for all strains (Gomand et al., 2018). Bacterial growth was monitored through OD_595 *nm*_ measurements over 48 h.

#### Data Processing

##### Strain growth comparison

The times at which the apparent bacterial growth starts (t_start_) were monitored such as described by Gomand et al. (2018). The higher these time values are, the later the growth starts i.e., the fewer bacteria have adhered i.e., the lower the affinity. These values were averaged on all series of experiments and standard deviations are computed. Strains were compared to one another based on their minimum adhesion value (MAV) corresponding to the difference between the smallest *t*_start_ (highest adhesion) obtained on a control without β-lactoglobulin and the highest t_start_ (lowest adhesion) obtained on β-lactoglobulin:

$$\mathrm{Minimum adhesion value}\;(\text{MAV})={\left(\left[{\left(t_{\text{start}}\right)}_{\text{average}}\right]-\sigma \right)}_{\mathrm{Control}}-{\left(\left[{\left(t_{\text{start}}\right)}_{\text{average}}\right]-\sigma \right)}_{\beta -\text{lactoglobulin}}$$

where σ stands for standard deviation. A strain is considered to adhere to β-lactoglobulin if its MAV is significantly superior to zero for all three series of experiments.

##### Functional domain prediction for the bacterial surface proteome

Bacterial surface proteins featuring LPxTG motif were predicted using the InterPro resource, that provides functional analysis of protein sequences by classifying them into families and predicting the presence of domains and important sites (Finn et al., 2017). Protein sequences were obtained from Sun et al. (2015) and were scanned against InterPro’s signatures using the software package InterProScan (Jones et al., 2014). Gene sequence resemblance with known domains was performed using the Basic Local Alignment Search Tool resource (BLAST), according to Altschul et al. (1990).

##### Statistical analysis

Statistical analysis were performed via *t*-tests and Tukey tests (parametric) for normal data and Wilcoxon–Mann Whitney and Steel-Dwass tests (non-parametric) for data that did not fit normal distribution using Kyplot software (Kyens Lab Inc.).

### Adhesive Interactions Between Bacteria and β-Lactoglobulin Characterized Through Atomic Force Microscopy

Protocols used in this part have been adapted from Guerin et al. (2018a). Briefly, this method consists in immobilizing the bacterial strains of interest on functionalized gold-coated mica by deposing the bacterial suspension during 15 h at 4°C (pH 6.8). The mica is rinsed with PBS (pH 6.8) before use. Milk proteins are prepared in distilled water (1% w/w) and adsorbed on modified AFM probes (gold coated and with NH2-terminated PEG linker) by immersion for 15 h at 4°C and then rinsed with milli-Q-grade water before use. Force measurements are performed at room temperature in PBS buffer (pH 6.8). AFM force distance curves are obtained by following the cantilever deflection as a function of the vertical displacement of the piezoelectric scanner with a scan speed of 400 mm/s. Adjustments to this protocol are listed below.

#### Bacterial Cultures

Cultures were prepared according to Guerin et al. (2018a). Precultures of *Lactobacillus aquaticus* DSM 21051 and *Lactobacillus sharpeae* DSM 20505 were prepared by inoculating 9 mL of MRS broth with 100 μL of bacterial stock and grown overnight at 37°C. These precultures were used to inoculate 9 mL of fresh MRS broth the next day and the growth was performed at 37°C until an optical density of 1.2 was reached at 660 nm (for about 8 h). Cultures were then centrifuged at 3,000 × *g* for 10 min at room temperature. Pellets were suspended in 1 mL of PBS (pH 6.8).

#### Preparation of Bacteria-Coated Mica and Protein-Coated Tips

According to Guerin et al. (2018a), a mica coated with a gold layer functionalized with a NH_2_-terminated PEG-linker (Novascan, Ames, IA, United States) was used, as well as AFM probes with borosilicate glass particle (2 μm), coated with gold and modified with NH_2_ terminated PEG linker (Novascan, Ames, IA, United States). The bacterial suspension is deposed on mica at 4°C and left overnight (pH 6.8). Preparation of the β-lactoglobulin and Bovine Serum Albumine (BSA) 1% (w/w) solutions (Sigma-Aldrich Co. LLC, St. Louis, MO, United States) was done according to Guerin et al. (2018a). Probes tips were left to incubate overnight at 4°C in wells containing 1 mL of the β-lactoglobulin or BSA solutions to maximize protein adsorption. β-lactoglobulin was the candidate protein tested and BSA was the negative control.

#### AFM Measurements

Protocol followed is described by Guerin et al. (2018a). Force-volume measurements are performed at room temperature in PBS buffer (pH 6.8) using a Bruker Bioscope Resolve atomic force microscope (Bruker Corporation, Santa Barbara, CA, United States) mounted on an inverted microscope (DMi8, Leica Microsystems). The spring constants of the cantilevers was measured using the thermal noise method and found to be 0.01 N m^−1^. Force distance curves were recorded between the bacteria deposited on functionalized mica and the probe coated with β-lactoglobulin or BSA. Three adhesion force maps (20 μm × 20 μm, 256 force curves) were recorded for each protein-bacteria interaction analysis. Data analysis was performed using the Nanoscope Analysis software from Bruker (Santa Barbara, CA, United States) and the last peak was calculated for each curve before plotting adhesion forces and last rupture length histograms. The last peak is used for analysis instead of the maximum peak in order to characterize the last interacting point between the β-lactoglobulin and the cell receptor and not the unfolding of a biomolecular domain.

### Adhesive Interactions Imaged by Confocal Microscopy

The cultures were prepared as described in Section “Bacterial Cultures”, then centrifuged at 3,000 × *g* for 10 min at room temperature. Pellets were suspended in 10 mL of WPI solution (15%, w/w). The WPI solution was prepared using PRODIET 90 S (Ingredia, Arras, France) that is a soluble milk protein isolate containing native whey proteins including β-lactoglobulin. One milliliter of resuspended cells was stained with the LIVE/DEAD BacLight viability kit (1:200 v/v; LIVE/DEAD BacLight viability kit was prepared according to the procedure described for the kit L13152 by Thermo Fisher Scientific). Two hundred microliters of LAB suspension (same conditions as in Section “Bacterial Cultures”) were introduced on chambered glass slides (Nunc Lab-Tek, Thermo Fisher Scientific). CLSM images were taken using a Leica TCS SP5-X-AOBS confocal laser scanning microscope (Leica Microsystems CMS GmbH, Mannheim, Germany) equipped with WLL lasers. The objective lens used was a HCX PL APO CS 100 × 1.40 (oil immersion). The excitation wavelength was 488 nm and emission bandwidth was of 495–510 nm for SYTO 9 and 600–620 nm for propidium iodide. Two independent repetitions were performed and approximately 20 representative images were acquired for each sample.

## Results

### Identification of Strains Adhesive to β-Lactoglobulin

Most strains were found not to be adhesive to β-lactoglobulin as the average MAV calculated on the 73 strains was negative (−180 ± 22) although higher than the MAV of the negative control LGG *spaCBA* (−386), known to be non-adhesive to β-lactoglobulin (Guerin et al., 2016). The microplate adhesive assays revealed four adhesive candidates to β-lactoglobulin amongst the 73 strains tested: *L. aquaticus* DSM 21051 (MAV = 61.5), *Lactobacillus murinus* DSM 20452 (MAV = 12.8), *Lactobacillus plantarum* DSM 13273 (MAV = 12.6), *Lactobacillus brantae* DSM 23927 (MAV = 6.97), although these strains were still less adhesive than the positive control LGG WT (MAV = 104). Nine strains were also found to have a MAV inferior to the one of the negative control LGG *spaCBA*: *Lactobacillus sharpeae* DSM 20505 (MAV = −857), *Lactobacillus kefiri* DSM 20587 (MAV = −787), *Lactobacillus similis* DSM 23365 (MAV = −780), *Lactobacillus pobuzihii* DSM 28122 (MAV = −617), *Lactobacillus namurensis* DSM 19117 (MAV = 516), *Lactobacillus satsumensis* DSM 16230 (MAV = −490), *Pediococcus parvulus* DSM 20332 (MAV = −477), *Lactobacillus senmazukei* DSM 21775 (MAV = −404), *Lactobacillus lindneri* DSM 20690 (MAV = −387). The MAV for all strains are listed as Supplementary Data S1.

### Biophysical Deciphering of Bacterial Adhesive Interaction With β-Lactoglobulin Through AFM

**FIGURE 1:**
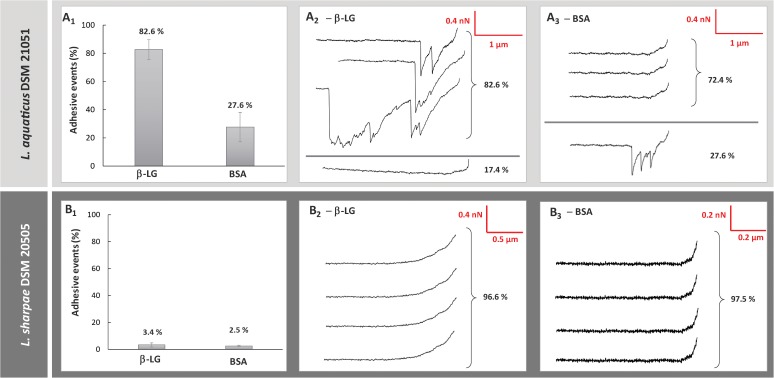
Comparison of the adhesive properties of two strains (*Lactobacillus aquaticus* DSM 21051, *Lactobacillus sharpeae* DSM 20505) for whey proteins isolates probed by atomic force microscopy (AFM): frequency of adhesive events occurring between whey proteins and *L. aquaticus* DSM 21051 **(A_1_)** and *L. sharpeae* DSM 20505 **(B_1_)** and representative examples of retraction curves obtained for force measurements between *L. aquaticus* DSM 21051 and β-lactoglobulin **(A_2_)**, *L. aquaticus* DSM 21051 and BSA **(A_3_)**, *L. sharpeae* DSM 20505 and β-lactoglobulin **(B_2_)**, and *L. sharpeae* DSM 20505 and BSA **(B_3_)**.

**FIGURE 2:**
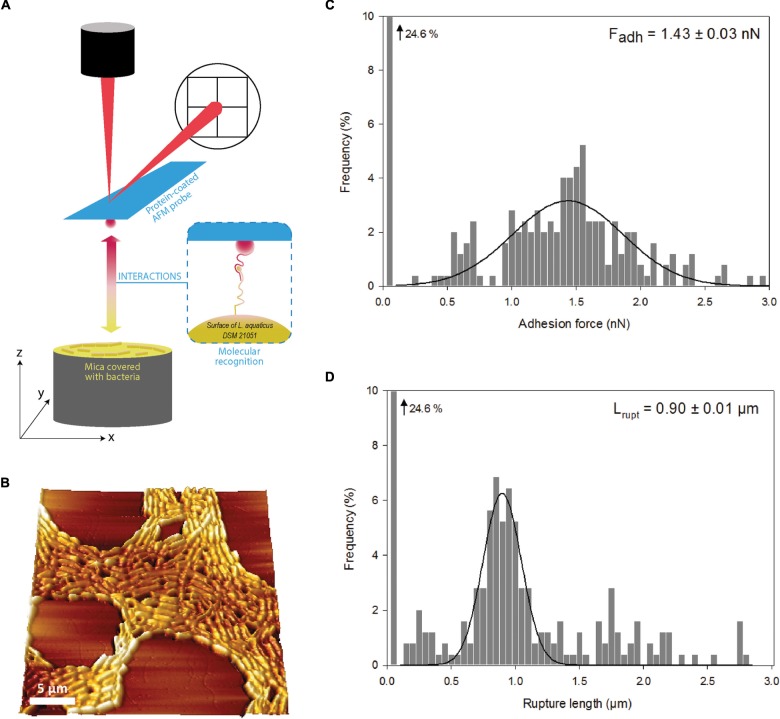
Schematic description of atomic force microscopy (AFM) with protein-coated tips and bacteria-coated mica. **(A)** 3D-AFM image of *Lactobacillus aquaticus* DSM 21051 recorded in liquid in phosphate buffered saline. **(B)** Interactions between β-lactoglobulin and *L. aquaticus* DSM 21051 explored by force measurement using AFM: adhesions forces **(C)** and final rupture length **(D)**. Averages of adhesion forces and rupture lengths are precised in panels **(C)** and **(D)** with standard errors.

The adhesive interactions between β-lactoglobulin and the strains at the extremes of the adhesion spectrum, *L. aquaticus* DSM 21051 (the most adhesive strain) and *L. sharpeae* DSM 20505 (the least adhesive strain) were studied through AFM, in order to characterize them in further depth. Only two strains were chosen to precise our understanding of the interaction mechanism of the LAB surface with β-lactoglobulin since AFM is not a suitable method for screening of large populations. This is why we decided to select only the two strains at the extreme of the adhesion spectrum for this analysis. BSA was used as a negative control as LAB strains have previously been found to feature low adhesion to it (Guerin et al., 2016; Gomand et al., 2018). The percentages of adhesive events (frequencies) observed between *L. aquaticus* DSM 21051 and the two proteins, β-lactoglobulin and BSA, were respectively of 82.6 ± 7.1% and 27.6 ± 10.4% (Figure 1A_1_). The frequencies of adhesive events observed between *L. sharpeae* DSM 20505 and the same two proteins were respectively of 3.4 ± 1.5% for β-lactoglobulin and 2.5 ± 0.6% for BSA (Figure 1B_1_). Typical force-distance curves obtained for the interactions occurring between the two strains and the AFM probes functionalized with the two proteins are presented, i.e., *L. aquaticus* DSM 21051 and β-lactoglobulin (Figure 1A_2_), *L. aquaticus* DSM 21051 and BSA (Figure 1A_3_), *L. sharpeae* DSM 20505 and β-lactoglobulin (Figure 1B_2_), and *L. sharpeae* DSM 20505 and BSA (Figure 1B_3_). During the withdrawal of functionalized β-lactoglobulin-coated probe from the surface of *L. aquaticus* DSM 21051 several specific adhesive events occur (Figure 1A_2_), whereas more than 70% of the curves observed for BSA-coated probes did not feature any adhesive event (Figure 1A_3_). Moreover, the few adhesive events observed between BSA and *L. aquaticus* DSM 21051 appeared to be random and therefore could not be associated to any specific interaction (Figure 1A_3_). Almost no adhesive event was observed for both BSA- and β-lactoglobulin-coated probes on *L. sharpeae* DSM 20505 cells (Figure 1B_1_–B_3_). These results are consistent with those obtained using the screening method: *L. aquaticus* DSM 21051 significantly adheres to β-lac whereas poor adhesion was observed for *L. sharpeae* DSM 20505. Retraction curves recorded between *L. aquaticus* DSM 21051 and β-lactoglobulin attest the specificity of occurring adhesive interactions, which would happen according to a lock and key mechanism (Figure 2A). 3D-AFM images recorded on mica attest of the good coverage of *L. aquaticus* DSM 21051 and therefore that adhesive events recorded did occur between *L. aquaticus* DSM 21051 cells and β-lactoglobulin-coated probes (Figure 2B). The biophysical properties of the adhesion between *L. aquaticus* DSM 21051 and β-lac were analyzed using additional force parameters including adhesion forces (Figure 2C) and final rupture length (Figure 2D). Retraction curves exhibited adhesion forces averaging around 1.43 ± 0.03 nN. Final rupture length averaged around 0.90 ± 0.03 μm. These results will be compared with those of LGG WT and the mutant strains LGG *spaCBA* and *welE* in the discussion section.

### Impact of Adhesive Interactions on Bacterial Distribution in Whey Protein Isolate Probed by Confocal Microscopy

**FIGURE 3:**
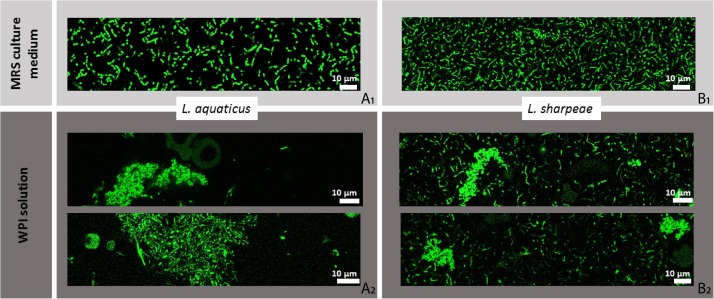
Spatial distribution of *L. aquaticus* DSM 21051 and *L. sharpeae* DSM 20505 in MRS culture medium **(A_1_,B_1_)** and in whey protein isolate (WPI) solution **(A_2_,B_2_)**, imaged by confocal laser scanning microscopy (CLSM). Bacterial concentration is 10^7^ u.f.c./mL. Bacteria cells are represented in green on this figure whether they are viable or damaged (no difference is made here that would depend on bacterial status).

**FIGURE 4:**
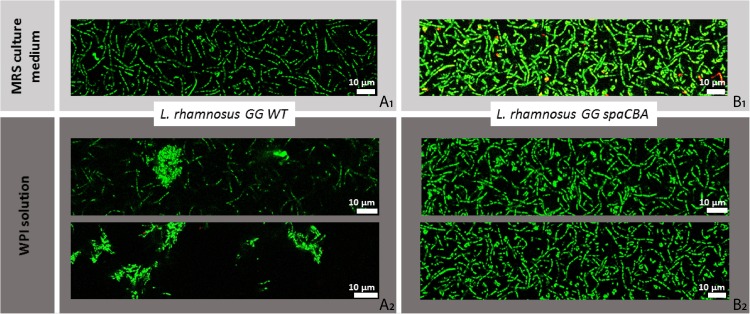
Spatial distribution of LGG WT and LGG *spaCBA* in MRS culture medium **(A_1_,B_1_)** and in whey protein isolate (WPI) solution **(A_2_,B_2_)**, imaged by confocal laser scanning microscopy (CLSM). Bacterial concentration is 10^7^ u.f.c./mL. Bacteria cells are represented in green on this figure whether they are viable or damaged (no difference is made here that would depend on bacterial status).

*Lactobacillus aquaticus* DSM 21051, *L. sharpeae* DSM 20505, LGG WT and LGG *spaCBA* were first imaged in MRS to make sure that they were originally homogeneously distributed (Figure 3A_1_,B_1_, 4A_1_,B_1_). Live cells of *L. aquaticus* DSM 21051 were found to aggregate in the WPI solution whereas *L. sharpeae* DSM 20505 live cells remained homogeneously distributed (Figure 3A_2_,B_2_). This is consistent with the adhesive properties of the control strains: LGG WT (positive control) aggregate in the WPI solution whereas LGG *spaCBA* remained homogeneously distributed (Figure 4A_2_,B_2_). Dead bacterial cells or cells with a damaged membrane gathered in flocs for all four strain types (data not shown).

### Relation Between Bacterial Adhesion to β-Lactoglobulin and Predicted Bacterial Surface Characteristics

#### Presence of Pilus Gene Clusters (PGCs)

Predicted bacterial surface characteristics were analyzed in relation to the results of the adhesive assays in order to delineate gene candidates predicted to encode surface proteins that could be involved in bacterial adhesion to β-lactoglobulin. Amongst the 73 strains tested, 32 of them possessed at least one sortase-dependent PGC and therefore were predicted to express pili on their surface (Sun et al., 2015). The average MAV of these 32 strains was −163 ± 33.2 whereas the average MAV of the 41 non-piliated strains was −194 ± 30.1. Amongst the 32 strains presenting PGCs, 16 possessed PGCs similar to LGG pilus clusters in terms of gene order, that is, a cluster of three pilin genes and one pilin-specific sortase gene (Sun et al., 2015). The MAV of these 16 strains was −165 ± 53.8 whereas the MAV of the 16 strains with PGCs different from LGG was −160 ± 38.8. Although a mean comparison of the MAV for strains featuring PGCs compared to non-piliated strains would suggest that the presence of PGCs fosters adhesion to β-lactoglobulin, this was not supported statistically. No difference could be observed between strains featuring PGCs similar to LGG WT’s and PGCs different from LGG WT’s. The number of PGCs, sortase enzymes or proteins with LPxTG motif (listed for all strains in S1) were not found either to impact strain adhesion to β-lactoglobulin (data not shown).

#### Predicted Protein Domains Candidates for Mediating Bacterial Adhesion to β-Lactoglobulin

More predicted surface characteristics were analyzed for the four strains found to be adhesive to β-lactoglobulin. Predicted protein domains featuring LPxTG motif found for each strain are listed in Table 1. Strains were analyzed for gene sequence resemblance with the *spaCBA* domain, known to be responsible for adhesion to β-lactoglobulin for LGG WT (Guerin et al., 2016) but no homologue sequence could be identified for any of the four adhesive strains. All strains are predicted to feature immunoglobulin-like (Ig-like) fold domains, which are usually involved in binding or molecular recognition processes (Bodelón et al., 2013). Other and more specific adhesion-related domains present on the four adhesive strains studied as well as on LGG WT include MucBP (mucin-binding), CBME/CBM3 (carbohydrate-binding), fibrinogen- and collagen-binding domains, cysteine- and leucine-rich domains, and SD-repeat B-domain. Most of these domains are present once in the genome of the adhesive strains (*L. plantarum* DSM 13273 is the only adhesive strain presenting three MucBP domains) and are not repeated within a given protein.

**Table 1:** Predicted proteins domains with LPxTG motif which may play a role in bacterial adhesion to β-lactoglobulin.

Strain			MAV	Predicted adhesion-related protein domains^∗^
*Lactobacillus aquaticus*	DSM	21051	61.5	**Immunoglobulin-like fold, MucBP**
*Lactobacillus murinus*	DSM	20452	12.8	**Immunoglobulin-like fold**
*Lactobacillus plantarum*	DSM	13273	12.6	**Immunoglobulin-like fold**, **MucBP**, CMBE-CBM3 (carbohydrate-binding), **fibrinogen-binding**, collagen-binding
*Lactobacillus brantae*	DSM	23927	6.97	**Immunoglobulin-like fold**, Collagen-binding surface protein Cna-like (type B), **Leucin-rich repeat, SD-repeat (type B)**
*Lactobacillus rhamnosus*	GG	WT	104	**Immunoglobulin-like fold**, **MucBP**, **Leucin-rich repeat**, Fn3-like (frequently found in the adhesin/invasin streptococcal C5a), gram-positive pilin subunit D1 N-terminal (containing ***spaCBA* domain, responsible of adhesion to β-lactoglobulin**)

*Domains present on L. rhamnosus GG (known to be adhesive to β-lactoglobulin) are included as a reference. Proteins sequences used were those provided by Sun et al. (2015). ^∗^Proteins containing-domains written in bold characters for the four adhesive strains are likely to mediate adhesive interactions with β-lactoglobulin.*

The MucBP domain is the only domain with a known adhesive-related function (apart from the Ig-like fold domain) which could be identified on *L. aquaticus* DSM 21051, the most adhesive strain to β-lactoglobulin. MucBP domains have been found predominantly in lactobacilli found naturally in intestinal niches, which suggests that they play an important role in establishing host-microbial interactions in the gut by binding mucus (Roos and Jonsson, 2002; Tassell and Miller, 2011). *L. plantarum* DSM 13273 is the strain featuring the highest number of adhesion-related domains in its genome (Table 1). This is also the only strain out of the four presenting fibrinogen- and collagen-binding domains. The fibrinogen-binding domain has been found to accommodate linear peptides with a certain degree of ligand sequence variability (Ponnuraj et al., 2003) and therefore might be able to interact with β-lactoglobulin. *L. brantae* DSM 23927 features leucine-rich repeats (LRRs) and SD-repeat (Sdr) domains (Table 1), both of them susceptible to play a role in adhesive interactions to β-lactoglobulin. LRRs have been found to provide a structural framework for the formation of protein-protein binding and interactions (Gay et al., 1991; Kobe and Kajava, 2001) and are likely to allow a broad range of ligands (Kobe and Kajava, 2001). Sdr-repeat domains are surface proteins that play an important role in *Staphylococcus aureus* adhesion and pathogenesis (McCrea et al., 2000; Wang et al., 2013). The protein containing Sdr-repeat domains may therefore be a good candidate for mediating adhesion to β-lactoglobulin for the strain *L. brantae* DSM 23927. No other adhesion-related domain than the Ig-like fold domain was identified on *L. murinus* DSM 20452 (Table 1), which would suggest that the protein containing this domain would likely be the one involved in adhesive interactions with β-lactoglobulin.

## Discussion

The aim of this study was to evaluate and characterize adhesive interactions occurring between LAB and β-lactoglobulin. A collection of 73 LAB strains was screened for their adhesive behavior toward β-lactoglobulin and strains at the extreme of the adhesion spectrum i.e., a highly adhesive and a poorly adhesive strains were studied in further depth.

Only four strains out of 73 were found to present adhesive affinities toward β-lactoglobulin. Therefore, adhesion to β-lactoglobulin appears not to be a common characteristic of the LAB group. The consequences of these adhesive interactions, when they occur, are not fully understood. However, it could be hypothesized that strains featuring adhesive affinities toward whey proteins would be lost during the drainage step of cheese manufacturing processes, alongside with whey expulsion from the cheese network. It would be interesting to test the affinity of this same strain collection to other food components in future work, in order to dispose of more comparison points to our study and to get a better understanding of the importance of adhesion to β-lactoglobulin compared to adhesion to other food components. Currently, the rare existing studies discussing bacterial adhesion to food components other than β-lactoglobulin concern up to four strains at most at a time (De Bellis et al., 2010; Chumphon et al., 2016; Tarazanova et al., 2017, 2018a,b; Utratna et al., 2017), therefore failing to provide an overview of adhesion to food components amongst wide bacterial groups such as the LAB group.

The study performed by Tarazanova et al. (2017) is the only one to our knowledge that compares the adhesion level of a wide number of strains (55) to food (casein-derived) components, however these strains are all of the same species, *L. lactis*. Out of 55, 30–40 strains presented adhesive affinities toward casein-derived components, depending on their growth phase, and strains isolated from a dairy environment presented much stronger binding of milk proteins versus strains isolated from plants, suggesting a selective advantage (Tarazanova et al., 2017). However, this was not confirmed in our case, as the four strains out of 73 that were originally isolated from dairy products, i.e., *Lactobacillus casei* DSM 20011, *L. paracasei* subsp. *tolerans* DSM 20258, *Lactobacillus bifermentans* DSM 20003, and *L. kefiri* DSM 20587, did not present more adhesive affinities toward β-lactoglobulin in average than the strains isolated from nondairy sources (data not shown).

The strain found to be the most adhesive to β-lactoglobulin, *L. aquaticus* DSM 21051, exhibited a specific adhesive behavior when studied by AFM. The signature of the observed retraction curves was identified as specific of biomolecules stretching, suggesting that the surface of *L. aquaticus* DSM 21051 features a strong affinity toward β-lac. This has also been shown previously for the model strain LGG WT by our team as well as for the mutant strain LGG *welE*, expolysaccharide-depleted and known to adhere more to β-lactoglobulin than LGG WT due to its increased pili exposure (Guerin et al., 2016, 2018a). *A contrario*, *L. sharpeae* DSM 20505 which screening results show not to adhere to β-lactoglobulin presented retraction curves characteristic of a lack of adhesion to β-lac when studied by AFM (frequency of adhesive events was inferior to 5%). Similarly, our team demonstrated previously this same fact for the model strain non-adhesive to β-lactoglobulin, LGG *spaCBA* (Guerin et al., 2016). Comparative results are presented in Table 2.

**Table 2:** Comparison of the adhesive capabilities of five strains to β-lactoglobulin when studied by atomic force microscopy: *L. aquaticus* DSM 21051, *L. sharpeae* DSM 20505, and the model strains LGG WT, LGG *spaCBA* (pili-depleted), and LGG *welE* (exopolysaccharides-depleted).

		Adhesive events (%)		Adhesion forces to β-lac (nN)	Length of the stretched biomolecule (μm)	References
		To β-lac	To BSA			
Strains highly	*L. aquaticus*	82.6 ± 7.1	27.6 ± 10.4	1.43 ± 0.03	0.90 ± 0.01	/
adhesive to β-lac	LGG WT	51.4 ± 9.9	13.1 ± 0.8	[0.13; 0.81] ± 0.01	0.39 ± 0.02	Guerin et al., 2016
	LGG *welE*	84.1 ± 3.0	88.5 ± 2.5	[0.58; 1.31] ± 0.01	0.93 ± 0.03	Guerin et al., 2016, 2017
Strains poorly	*L. sharpeae*	3.4 ± 1.5	2.5 ± 0.6	*NS*^∗^	/	/
adhesive to β-lac	LGG *spaCBA*	NS^∗^	/	*NS*^∗^	/	Guerin et al., 2016

*^∗^Frequency of adhesive events was found to be inferior to 5%.*

The adhesive behavior of *L. aquaticus* DSM 21051 toward β-lactoglobulin appears relatively close to the one of LGG *welE* in terms of frequency of adhesive events. The high specificity of the adhesion phenomenon occurring between *L. aquaticus* DSM 21051 and β-lactoglobulin is highlighted by the fact that the frequency of adhesion is almost twice as high as the one characterizing adhesive interactions between LGG WT and β-lactoglobulin, whereas the frequency of adhesion of *L. aquaticus* DSM 21051 on BSA is almost four times lower than the one occurring between LGG *welE* and BSA. The mean adhesion force recorded on the last peak is also three times higher than the mean adhesion force recorded for LGG WT and β-lactoglobulin, and higher than the highest adhesion force recorded on the last peak for LGG *welE* and β-lactoglobulin, reaffirming the idea of a very strong specificity and adhesion strength. When comparing the length of biomolecules stretched by adhesive interactions with β-lactoglobulin, *L. aquaticus* DSM 21051 and LGG *welE* both exhibit molecules stretched up to 1 μm i.e., three times longer than the molecule stretched in the case of LGG WT (Table 2). The molecule mediating adhesive interactions with β-lactoglobulin in the case of *L. aquaticus* DSM 21051 is therefore comparable in length to LGG pili when stretched, which may explain the higher specificity and adhesion strength found for *L. aquaticus* DSM 21051 compared to LGG WT, which pili are partially hidden within the exopolysaccharides layer (Guerin et al., 2016).

On the other hand, the frequency of adhesive events observed between *L. sharpeae* DSM 20505 and β-lactoglobulin is inferior to 5% and similar to the frequency of adhesive events observed on BSA for both this strain and *L. aquaticus* DSM 21051. The frequency of adhesive events recorded when using BSA-coated probes is also four times lower for *L. sharpeae* DSM 20505 than for LGG *spaCBA* (negative control). Overall, *L. sharpeae* DSM 20505 has demonstrated very poor adhesive capacities toward β-lactoglobulin. However, when analyzed for predicted adhesion-related protein domains, this strain revealed a total of 23 adhesion-related domains, 8 of which being different, including MucBP and gram-positive pilin subunit D1 N-terminal, although no sequence homologue to the *spaCBA* domain was found (data not shown). The *spaCBA* domain is known to mediate adhesion to β-lactoglobulin for the piliated strain LGG WT (Guerin et al., 2016). This confirms that adhesive interactions with β-lactoglobulin are specific, and cannot be predicted accurately using only genomic predictions (the functions of these domains may not be accurately predicted or they may not be expressed).

The gathering behavior observed by CLSM for the adhesive strains in the WPI solution also pledges in favor of a specific bacterial adhesion to β-lactoglobulin for *L. aquaticus* DSM 21051. CLSM results indicate that the location of bacteria in a dairy matrix strongly depends on bacterial surface properties. These observations are important as it was evidenced recently that physical properties of dairy products, such as viscosity and gel hardness, are affected by bacterial surface properties in the case of surface-engineered strains (Tarazanova et al., 2018a). In light of our results, it would be interesting to see if that is also the case for wild strains presenting different surface properties inducing different adhesive behaviors. Some peptides shown to be linked to bacterial aggregation were also recently evidenced to be able to promote bacterial adhesion to functionalized surfaces and Caco-2-cells (Okochi et al., 2017). This typical behavior was responsible for observed enhanced interactions between LAB and the host intestinal mucosa (Okochi et al., 2017). Adhesive interactions with β-lactoglobulin leading to the aggregation of *L. aquaticus* DSM 21051 and LGG WT cells might therefore be considered for further study in order to determine whether they would promote such kind of behavior as well.

This work was performed in the continuity of previous studies, in which a method was developed allowing screening a wide number of strains for their adhesive affinities toward biomolecules such as dairy food components (Gomand et al., 2018), and which identified the bacterial surface molecules (pili) involved in the adhesion of LGG to dairy components using AFM (Guerin et al., 2016). The present study sought to go beyond bacterial species differences in revealing common adhesive characteristics of LAB in relation to dairy food components such as β-lactoglobulin. We first looked for LAB species featuring adhesive affinities for β-lactoglobulin, then focused on the molecular characteristics of this adhesion. We observed adhesion to β-lactoglobulin for few LAB (less than 6% of our collection). However, for those which did feature adhesive affinities, some common characteristics were pointed out that matched the characteristics previously identified on the model strain LGG. These characteristics include the specificity of the affinity, as well as the impact on bacterial spatial distribution in the matrix. The major findings of the present paper are that (i) Adhesion to whey proteins is apparently not a common characteristic to the LAB group (few strains presented adhesive affinities toward β-lactoglobulin), (ii) Strains featuring adhesive affinities toward β-lactoglobulin present common adhesive characteristics (specific β-lactoglobulin-adhesion domains related to the specificity of the AFM signature), and (iii) Adhesion to β-lactoglobulin was shown to strongly influence bacterial distribution in dairy matrices featuring this component (adhesive bacteria gathered in flocs in whey matrices whereas non-adhesive bacteria distribute more homogeneously), and could therefore modulate their accessibility and later delivery when designing functional foods containing LAB with potential associated health effects.

According to these findings, food matrices could play a protective role on bacteria by influencing their spatial distribution, which may prove especially useful for probiotic bacteria. Indeed, as bacteria adhering to a component have been found to flocculate in the food matrix containing this component, this could result in later heterogeneous delivery in the gastro-intestinal tract (GIT) which would impact host colonization, but may also better protect bacterial survival until they reach the GIT. These findings also pave the road to future experiments aiming generalizing bacterial adhesion characteristics to broad bacterial groups, thus helping with practical food matrix design. It would therefore be interesting to study the potential protective effect of components to which bacteria are adherent during critical steps of the food manufacturing process, such as spray-drying during probiotic milk powder production.

## Author Contributions

FG, JG, JB, FB, and CG conceived the study. FG, JG, JB, SE-K-C, DD, and GF carried out the experiments. FG, JB, JG, SE-K-C, DD, and GF analyzed the data. FG, JG, and JB wrote the manuscript. All authors commented on the manuscript.

## Conflict of Interest Statement

The authors declare that the research was conducted in the absence of any commercial or financial relationships that could be construed as a potential conflict of interest.
